# Crude Turmeric Extract Improves the Suppressive Effects of *Lactobacillus rhamnosus* GG on Allergic Inflammation in a Murine Model of House Dust Mite-Induced Asthma

**DOI:** 10.3389/fimmu.2020.01092

**Published:** 2020-06-04

**Authors:** Fariba Ghiamati Yazdi, Amin Zakeri, Ingrid van Ark, Thea Leusink-Muis, Saskia Braber, Sabihe Soleimanian-Zad, Gert Folkerts

**Affiliations:** ^1^Department of Food Science and Technology, College of Agriculture, Isfahan University of Technology (IUT), Isfahan, Iran; ^2^Division of Pharmacology, Utrecht Institute for Pharmaceutical Sciences (UIPS), Faculty of Science, Utrecht University, Utrecht, Netherlands; ^3^Department of Clinical Medicine, Faculty of Health, Aarhus University, Aarhus, Denmark

**Keywords:** HDM-induced asthma, synbiotic, probiotic, *Lactobacillus rhamnosus* GG, allergic diseases, turmeric

## Abstract

There is a strong correlation between dysregulation of the gastrointestinal microbiota and development of allergic diseases. The most prevalent therapies for relieving asthma symptoms are associated with serious side effects, and therefore novel approaches are needed. Our objective was to elucidate whether oral administration of *Lactobacillus rhamnosus* GG (LGG) as a probiotic or turmeric powder (TP) as a prebiotic or both as a synbiotic mitigate allergic inflammation including lung function, airway inflammatory cell infiltration, Th2 cytokines/chemokine in a murine model of house dust mite (HDM)-induced asthma. BALB/c mice were intranasally sensitized and challenged with HDM received TP (20 mg/Kg mouse), or/and LGG (10^5^ or 10^7^ cfu/ml), or both orally. Interestingly, the synbiotic intervention (HDM-TP-LGG E7) specifically suppress the developement of airway hyperresponsiveness in response to methacholine. Besides, our synbiotic, TP, and LGG strongly down-regulated eosinophilia, IL-5, CCL17, IL-13. In terms of T cell response, CD4^+^ Th2 cells and CD4^+^ Th17 population were reduced in the splenocytes of the treatment groups compared to control. The synbiotic group not only elevated CD25^+^Foxp3^+^Treg frequency compared to asthmatic group, but also increased T reg cells compared to the probiotic group. The synbiotic also indicated the superior effect in suppressing Th2 cells compared to probiotic. Although, TP and LGG alone displayed suppressive effects, this study showed that the combination therapy consisting of TP and LGG (synbiotic) is more effective in some of the parameters than either of the treatments alone. This novel synbiotic, might be considered as a potential food-based drug for translational medicine and can possibly be used along with corticosteroid treatment.

## Introduction

Asthma is a complicated chronic disease of which the underlying immunological processes are still not well-grounded. According to the World Health Organization report, asthma is the most common non-communicable disease among children. It is estimated that about 235 million people currently suffer from asthma in which the majority of deaths occurs in the elderly. In terms of health economics, asthma has imposed a severe burden on the healthcare systems. There are many factors associated with the increasing frequency and severity of asthma, including genetic predisposition, allergen exposure, air pollution, and lifestyle. Allergic asthma is recognized by a dominant Th2 response causing insufficient lung function, airway inflammation, increased total IgE levels, and eosinophilia in bronchoalveolar lavage fluid (BALF) (1–3).

Currently, the most common therapies to control asthma symptoms are long-acting beta-agonists and corticosteroids. Substantial evidences show that long-term use of corticosteroids can cause corticosteroid-resistance leading to a poorly controlled disease. Besides, many side effects have been reported, including weight loss, growth reduction, increasing blood pressure, muscles, and bones atrophy. Thus, there is an urgent need for novel treatments with long-term persistence, stronger symptom alleviation, and minimum side effects (1, 2, 4, 5).

It is postulated that certain microorganisms and/or their metabolites can shift the inflammatory responses to Th1. On the other hand, they may augment the production of regulatory cytokines by proliferation of regulatory T cells (Treg). Interestingly, both levers (Th1 and Treg) can lead to downregulation of allergen specific Th2 responses. In this regard, the intestinal microbiota can play a central role in governing hyperactivation of cells toward balanced circumstance (2, 5, 6).

Probiotics are defined as the non-pathogenic microorganisms that award healthiness to host when are consumed in adequate numbers (6). They are able to affect both local (intestine) and systemic inflammation by secretion of several metabolites, like antimicrobial products or so-called “bacteriocin” and short chain fatty acid (SCFA) (7), selective enteric pathogen exclusion, stimulating intestinal tight junction network, and regulation of immunological responses (8).

Given the anti-inflammatory effect of probiotics, numerous studies have been conducted to evaluate their therapeutic effects on alleviating allergic asthma symptoms (9–14). Sagar et al. reported the promising effect of *Lactobacillus rhamnosus* and *Bifidibacterium animalis* bb12 on the reduction of lung resistance in the ovalbumin-induced allergic asthma in mice (1). Similarly, Wu et al. demonstrated that oral pre- and post-treatment of *Lactobacillus rhamnosus* GG not only decrease the lung resistance but also reduce BALF inflammatory cell filtration and Th_2_ cytokines in mice (2).

The growth, activity, and colonization of probiotic in GI can be stimulated by the use of prebiotics. Prebiotics are regarded as non-digestible food constituents consumed by probiotics. The synbiotic concept refers to the combination of pro- and prebiotics (11, 15, 16).

A growing body of clinical trials, epidemiological studies as well as animal experiments have described herbaceous medicines as novel complementary therapeutic modalities for many diseases (10, 17, 18).

Turmeric is a complex compound derived from the *Curcuma longa* rhizomes. According to our pervious study, the chemical analysis of turmeric extract indicated several components including curcumin (polyphenol yellowish pigment of turmeric 2–5%), carbohydrates (40–70%), proteins (6–8%), oils (5–8%), and other elements (3–5%) (19, 20). Turmeric has well-known pharmacological activities such as anti-inflammatory function. In addition, it has recently attracted attentions as a potential prebiotic compound (17, 19).

Although most animal models of asthma studies have focused on the effect of curcumin or probiotic bacteria alone, the effect of crude turmeric combined with a probiotic bacterium has poorly been addressed (19, 21). It was, therefore, the aim of this study to explore the effects of long-term treatment with *Lactobacillus rhamnosus* GG, turmeric powder, and their combination on airway inflammation. In order to induce an allergic asthma model, mice were sensitized and challenged with house dust mite (HDM) Pro-, pre-, and synbiotic were administrated orally and compared to budesonide as a standard therapy.

The present study indicates that a novel synbiotic can potentially synergize the protective effects of probiotic and prebiotic in context of allergic airway inflammation via suppression of airway hyperresponsiveness (AHR) BALF eosinophilia, and Th2 cells and associated cytokines accompanied by induction of regulatory T cells.

## Materials and Methods

### Mice

Male BALB/c mice at 6–8 weeks of age with body weights of 20–25 g were purchased from the Charles River Laboratories, France and were acclimatized for 1 week prior to the start of the experiments. Mice were housed in filter-topped makrolon cages (Plexx, The Netherlands) and had free access to food and water. The AIN93G control diet (Research Diet Services, The Netherlands) was used to feed the mice *ad-libitum* during the entire experimental period. All *in vivo* experiments were performed in accordance with the Guidelines of the Dutch Committee of Animal Experiments (Utrecht, the Netherlands).

### The Probiotic and Prebiotic

Lactobacillus rhamnosus GG-ATCC 53103 (LGG) was purchased from the American Type Culture Collection (USA). A capsule of LGG was inoculated in MRS broth (Oxoid, UK) at 37°C overnight and bacteria were harvested in the late logarithmic phase by centrifugation (3,200 g, 10 min), washed with phosphate buffered saline (PBS, Dulbecco's phosphate-buffered saline, Sigma) twice and counted by spectroscopy method (optical density) and plating serial dilutions (19, 21). According to the growth curve obtained from daily monitoring of Lactobacillus rhamnosus GG-ATCC 53103, active fresh bacteria prepared daily in the sterile PBS for oral treatments in two different doses (5 × 10^5^ cfu/ml, 200 μl and 5 × 10^7^ cfu/ml, 200 μl).

Rhizomes of *Curcuma longa* plant (turmeric) used in this study were provided from the local markets of Isfahan province, Isfahan, Iran. The rhizomes were peeled, chopped and blended with a miller (HR 2061; Philips, Netherland), and being passed through a sieve (mesh number 140,105 μm hole size) to make turmeric powder (TP) for further experiment. TP was dissolved in PBS to achieve an appropriate concentration. The solution was sterilized by autoclave to prevent microbial contamination.

### Murine HDM-Induced Asthma Model

**Figure 1:**
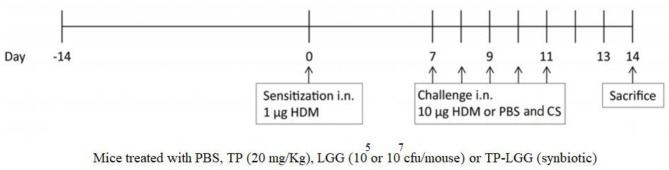
Schematic overview of the experimental protocol. BALB/c mice (*n* = 6/group) were intranasally sensitized (i.n.) with house dust mite (HDM) or PBS on day 0 and were challenged i.n. for five consecutive days (7–11) with HDM or PBS. The oral gavage treatment started 2 weeks prior to sensitization. By oral gavage, the mice were received 20 mg/kg TP (prebiotic), or 10^5^ or 10^7^ cfu/mouse LGG (probiotic) or turmeric in combination with 10^5^ or 10^7^ cfu/mouse of LGG (synbiotic) once a day, throughout the study. The mice were sacrificed on day 14.

BALB/c mice were intranasally sensitized on day 0 with 1 μg HDM (Greer Laboratories, Lenoir, USA)/40 μL PBS (Lonza, Walkersville, USA) or PBS alone under the mild anesthetic circumstance induced by isoflurane inhalation. The protocol was followed by intranasal challenges once a day from day 7 to 11 with 10 μg HDM/40 μL PBS or PBS alone (HDM-PBS = positive control groups or PBS-PBS = negative control groups) (Figure 1 and Table 1) (1, 3, 4, 22). The test groups include: PBS-PBS (control negative group): PBS sensitized, challenged and treated mice, PBS-TP: The control of prebiotic (TP) group was sensitized and challenged with PBS and orally treated once a day with TP (20 mg/kg) (17), PBS-TP- LGG E7: The synbiotic control group was sensitized and challenged with PBS and treated (oral gavage) with a 200 μl mixture of TP (20 mg/kg) and 10^7^ cfu LGG /mouse HDM-PBS (control positive group): HDM sensitized and challenged, and PBS treated mice, HDM-CS: As a treatment positive control, intratracheal instillation from day 7 till 11 with the corticosteroid budesonide (CS) as HDM-CS group (500 μg/kg, 40 μl), the prevalent therapy for asthmatic patients, was performed once a day to determine the effectiveness of our treatments. HDM- LGG E5: The probiotic group 1 was sensitized and challenged with HDM and treated (oral gavage) with 200 μl of 10^5^ cfu/mouse LGG, HDM-LGG E7: The probiotic group 2 was sensitized and challenged with HDM and treated (oral gavage) with 200 μl of 10^7^ cfu/mouse LGG (Figure 1 and Table 1) (1, 3, 4, 22) HDM-TP: The prebiotic (TP) group was sensitized and challenged with HDM and orally treated once a day with TP (20 mg/kg), HDM-TP-LGG E5: The synbiotic group 1 was sensitized and challenged with HDM and treated (oral gavage) with a 200 μl mixture of TP (20 mg/kg) and 10^5^ cfu LGG /mouse, HDM-TP-LGG E7: The synbiotic group 2 was sensitized and challenged with HDM and treated (oral gavage) with a 200 μl mixture of TP (20 mg/kg) and 10^7^ cfu LGG /mouse. The oral treatments started 2 weeks before sensitization (day 0) and were continued throughout the entire procedure (day 11). Mice were sacrificed on day 14 by pentobarbital overdose (Figure 1 and Table 1) (1, 3, 4, 22).

**Table 1:** Summary of experimental platform grouping different conditions.

**Group name**	**Sensitization HDM = 1 μg/40 μl i.n. ^a^ = 40 μl PBS = 40 μl**	**Challenging i.n. = 40 μl**	**Oral treatment (from day-14 to day 14)) oral gavage = 200 μl)**	**CS treatment budesonide 0.5 mg/kg i.t. ^b^ = 40 μl**
PBS-PBS	PBS	PBS	PBS	-
HDM-PBS	HDM	HDM	PBS	-
HDM-CS	HDM	HDM	PBS	Budesonide 0.5 mg/kg i.t. = 40 μl
PBS-TP-LGG7	PBS	PBS	TP 20 mg/kg and LGG E7 cfu/ml	-
HDM-TP-LGG7	HDM	HDM	TP20 mg/kg and LGG E7 cfu/ml	-
HDM-TP-LGG5	HDM	HDM	TP20 mg/kg and LGG E5 cfu/ml	-
PBS-TP	PBS	PBS	TP 20 mg/kg	-
HDM-TP	HDM	HDM	TP 20 mg/kg	-
HDM-LGG E5	HDM	HDM	LGG E5 cfu/ml	-
HDM-LGG E7	HDM	HDM	LGG E7 cfu/ml	-

a *i.n., Intranasal*.

b *i.t., intratracheal*.

### Airway Responsiveness Measurement

On day 14, mice underwent anesthesia by intraperitoneal injection of a K-M-mixture containing ketamine (Vetoquinol S.A., France; 125 mg/kg, i.p.) and medetomidine (Pfizer, The Netherlands; 0.4 mg/kg, i.p.). The lung resistance to the increasing doses of methacholine (acetyl-β-methyl-choline chloride, Sigma-Aldrich, The Netherlands; 0–25 mg/mL, 10% puff/10 sec.) was measured by EMKA invasive measuring instrument of dynamic lung resistance and compliance (EMKA Technologies, France) (1, 3).

### Serum

The lung function measurement was carried out on day 14. Blood was collected by cardiac puncture for measuring total serum IgE and mice were sacrificed with the overdose of pentobarbital injection intraperitoneally (600 mg/kg, Nembutal™, The Netherlands). The blood was coagulated for 30 min at room temperature and centrifuged at 13,300 rpm for 5 min. Serum samples were stored at −20°C until further use.

### Bronchoalveolar Lavage

Lungs were gently lavaged with 1 ml of pre-warmed pyrogen-free saline (0.9% NaCl, 37°C) containing protease inhibitor tablet (Complete Mini, Roche Diagnostics, Germany). The first lavage containing inflammatory cells was used for cytokines and chemokines measurements. Three additional lavages with 1 ml of saline were carried out to maximize cell harvesting from the BALF. The BALF cells obtained from the 4 times lavaging, were centrifuged (400 g, 5 min), and the pellets were pooled. The total numbers of cells were counted by the use of Bürker-Türk chamber. Afterward, cytospin preparations were made (centrifugation 20 g, 5 min, 4°C onto the glass) and stained by Diff-Quick method (Merz & Dade A.G., Switzerland) to differentiate BALF cell counts. The number of macrophages, eosinophils, neutrophils, and lymphocytes was determined by light microscopy (1, 3).

### Preparation of Lung Homogenates

The lungs were homogenized into the Precellys 24 tissue homogenizer tubes (Bertin Technologies, France) which contained 500 μl of 1% Triton X-100 (Sigma-Aldrich) and PBS containing protease inhibitor (Complete Mini, Roche Diagnostics, Germany). The sample solutions were homogenized by using the homogenizer instrument three times for 10 s. at 6,000 rpm with a minimum of 2 min interruption period for cooling in between. The supernatant was collected, centrifuged at 14,000 rpm for 10 min and stored at −20°C for further experiments. Pierce BCA protein assay kit was used to determine the protein concentration of each sample according to the manufacturer's protocol (Thermo Fisher Scientific, USA). The homogenized samples were normalized to the concentration of 1 mg protein/ml (3, 4).

### Cytokines Measurement

The supernatants of the lung homogenates were assayed for the determination of cytokines and chemokine. IL-33 and CCL17 were measured via the package of DuoSet ELISA (R&D Systems) and IL-13, IL-5, and total IgE were measured with a Ready-SET-Go! ® ELISA (eBioscience^TM^ (IL13, total IgE) and Invitrogen^TM^ IL-5, USA) kit. All ELISA assays were performed according to the manufacturer's protocol. The concentrations of the measured cytokines and chemokines were expressed as pg/mg protein in lung homogenates and pg/mL in serum. The ELISA plates were read at 450 nm using a Bio-Rad ELISA Reader (Bio-Rad, Hercules, CA, USA) (3, 4).

### Flow Cytometric Analysis of Immune Cells in the Spleen

Splenocyte cell suspensions were resuspended in PBS blocking buffer containing 1% BSA and 5% FCS (Sigma-Aldrich) and incubated for 15 min at 4°C with Fc block CD16/CD32 antibodies (BD Biosciences; 5 μg/mL) to prevent non-antigen-specific binding. Cells (5 × 10^5^) were subsequently stained with antibodies (eBioscience, The Netherlands, unless otherwise stated) against CD69-APC, CD4-PerCP Cy5.5, CXCR3-PE, T1ST2-FITC, RORγ-APC, CD25-Alexa Fluor® 488, FoxP3-PE Cy7, CD196-PE, and Fixable Viability Dye-eFluor® 780 (eBioscience, USA) or matching isotype controls for 30 min at 4°C. Cells were fixed using fixation buffer (eBioscience) or permeabilized for intracellular staining using the intracellular staining buffer set (eBioscience) according to the manufacturer's protocol. Flow cytometry was performed using FACS Canto II (BD Biosciences), and results were analyzed using Flowjo Software V. 10.6.2 (Becton Dickinson & Company (BD). We used fluorescence minus one (FMO) to differentiate between negative and positive staining cell populations (3, 22).

### Statistical Analysis

Results were represented as mean ± standard error of the mean (SEM). Data were statistically analyzed using a one-way ANOVA and *post-hoc* Bonferroni's multiple comparisons test. Significance limits were set at *p* ≤ 0.05. Statistical analysis was conducted using Graph Pad Prism software (version 7.04, Graph Pad Software, Inc.) (19, 22).

## Results

### Oral Administration of LGG (Probiotic), TP (Prebiotic), and LGG-TP (Synbiotic) Reduced Airway Hyperresponsiveness in HDM-Allergic Mice

**Figure 2:**
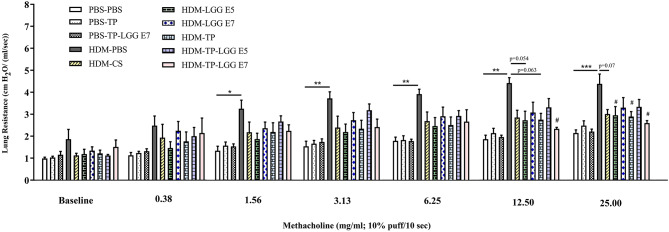
House dust mite (HDM)-induced airway resistance diagram. The airway resistance was abrogated upon oral administration of 20 mg/kg TP (prebiotic), or 10^5^ or 10^7^ cfu/mouse LGG (probiotic) or TP in combination with 10^5^ or 10^7^ cfu/mouse of LGG (synbiotic). Lung resistance (RL) measured in response to increasing doses of methacholine. PBS-PBS (control negative group): PBS sensitized, challenged, and treated mice, PBS-TP: PBS sensitized and challenged, and TP (20 mg/kg) treated mice, PBS-TP- LGG E7: PBS sensitized and challenged, and synbiotic (with 10^7^ cfu/mouse LGG) treated mice, HDM-PBS (control positive group): HDM sensitized and challenged, and PBS treated mice, HDM-CS: HDM sensitized and challenged, and corticosteroid treated mice, HDM- LGG E5: HDM sensitized and challenged, and 10^5^ cfu/mouse probiotic treated mice, HDM-LGG E7: HDM sensitized and challenged, and 10^7^ cfu/mouse probiotic treated mice. HDM-TP: HDM sensitized and challenged, and prebiotic treated mice, HDM-TP-LGG E5: HDM sensitized and challenged, and synbiotic (with 10^5^ cfu/mouse LGG) treated mice, HDM-TP-LGG E7: HDM sensitized and challenged, and synbiotic (with 10^7^ cfu/mouse LGG) treated mice. Results are shown as mean ± *SEM*. *P < 0.05, **P < 0.01, and ***P < 0.001 compared to PBS-PBS group, and ^#^*P* < 0.05 compared to HDM-PBS group as analyzed using One-Way ANOVA and *post-hoc* Bonferroni's multiple comparisons test. *n* = 6 mice/group.

The EMKA lung function system was applied to detect airway hyperresponsiveness (AHR). The baseline resistance (0.90 ± 0.05 cm H_2_O/(ml/sec) in PBS-PBS (negative control group) was similar between the experimental groups (Figure 2). Methacholine concentration-dependently increased airway resistance and was significantly enhanced in the HDM-PBS group [RL 1.87 ± 0.06 to 4.38 ± 0.05 cm H_2_O/(mL/sec)] compared to PBS-PBS group. Lastly, 1.56 mg/mL of methacholine caused a significant difference between PBS-PBS and HDM-PBS groups (p < 0.05). The airway hyperresponsiveness of the mice receiving either 20 mg/kg TP (HDM-TP) or 10^5^ cfu/mouse of LGG was significantly decreased at 25 mg/mL of methacholine (Figure 2), while the synbiotic (HDM-TP-LGG E7) seems to be the most effective and started to alleviate hyperresponsivity from lower concentration of methacholine (12.5 mg/mL) (Figure 2). A consistent decrease was observed in the HDM-CS group although the values did not reach statistical significance (Figure 2).

### Oral Administration of LGG, TP, and LGG-TP Reduced Inflammatory Cells in BALF of HDM-Allergic Mice

**Figure 3:**
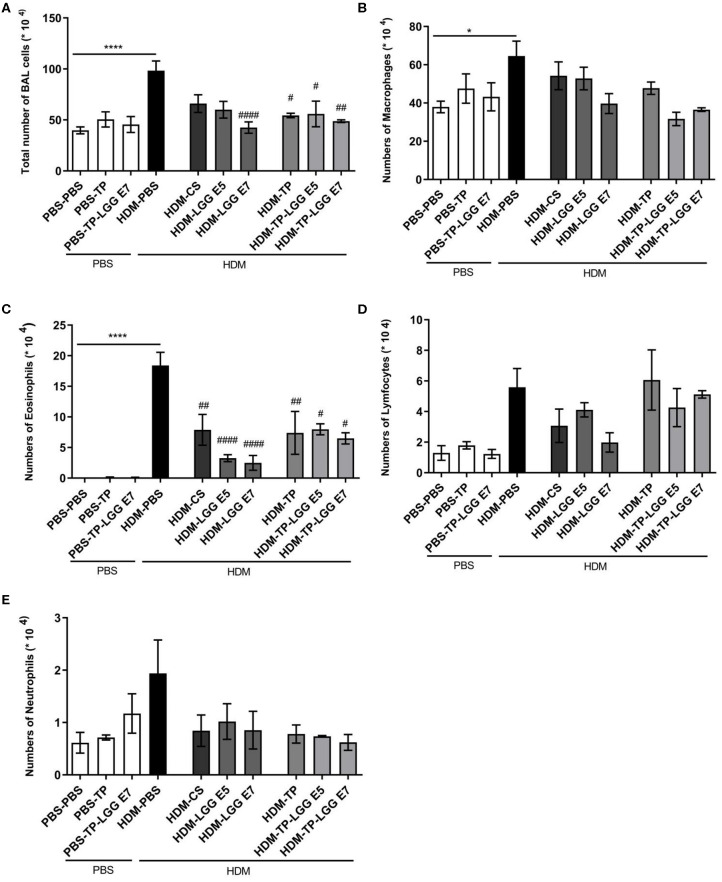
Differential inflammatory cell count of bronchoalveolar lavage fluid (BALF). **(A)** Total BALF cells, **(B)** the absolute number of macrophages, **(C)** eosinophils, **(D)** lymphocytes and, **(E)** neutrophils. Data are shown as mean ± *SEM, n* = 6 mice/group. *P < 0.05 and ****P < 0.0001 compared to PBS-PBS group, ^#^*P* < 0.05, ^##^*P* < 0.01, and ^####^*P* < 0.0001 compared to HDM-PBS group. Statistical significance of differences was tested by use of One-Way ANOVA and *post-hoc* Bonferroni's multiple comparisons test.

Accumulation of inflammatory cells in the lung occurs as the result of airway inflammation in asthma. The bronchoalveolar lavage fluid (BALF) was analyzed regarding the inflammatory cell influx into the airways of control and treatment groups (Figure 3). The total number of BALF cells was increased in the mice sensitized with HDM (HDM-PBS) compared to PBS-PBS group (Figure 3A). This increase was mainly due to eosinophils (Figure 3C) compared to the control group (p < 0.05). The same trend was observed for macrophages (Figure 3B), neutrophils (Figure 3E) and lymphocytes (Figure 3D) but it was not statistically significant. Importantly, the probiotic, prebiotic, and synbiotic intervention significantly reduced the cell infiltration in the BALF (p < 0.05 which was mainly due to a reduction in eosinophils (2, 3). The number of eosinophils was significantly reduced in the HDM-CS group (p < 0.05).

### Effect of Oral Administration of LGG, TP, and LGG-TP on the Attenuating of Th2-Type Mediators in Lungs and IgE Levels in Serum of HDM-Allergic Mice

The Th_2_ cytokines that play an important role in allergic asthma, like IL-5, IL-13, CCL17, and IL-33 along with total IgE in serum were measured. The mentioned cytokines and chemokine were significantly increased in the lung homogenates of HDM–PBS compared to the PBS–PBS control group.

**Figure 4:**
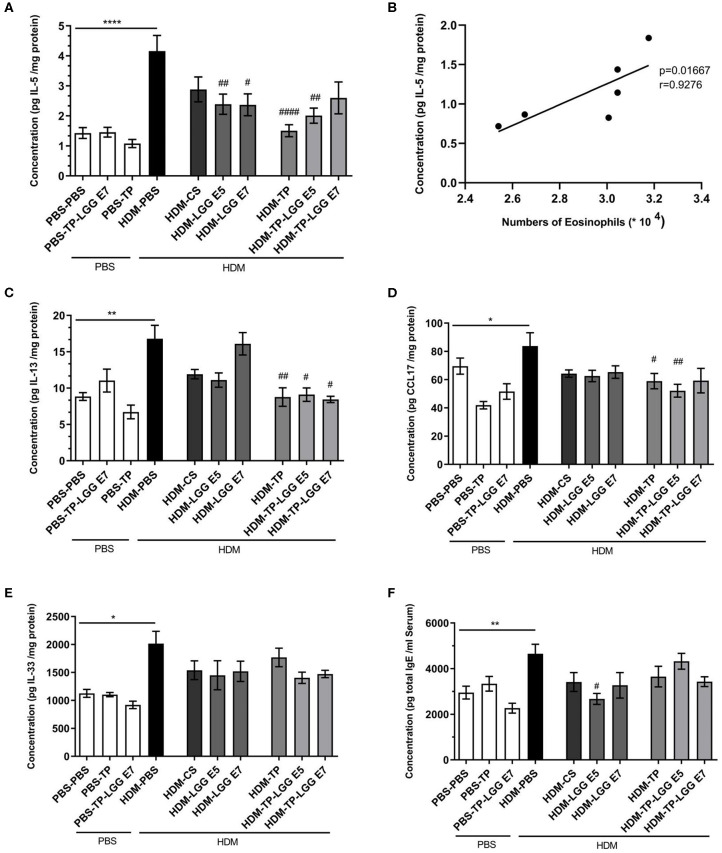
Immune-related mediators' measurements. IL-5, IL-13, IL-33, CCL17, and total IgE concentrations were measured in supernatant of lung homogenates (pg/mg protein) and in serum for Total IgE in HDM-allergic mice. IL-5 **(A)**, IL-5-eosinophils correlation **(B)**, IL-13 **(C)**, CCL17 **(D)**, IL-33 **(E)**, and total IgE **(F)**. Data are shown as mean ± *SEM, n* = 6 mice/group. *P < 0.05, **P < 0.01, and ****P < 0.0001 compared to PBS-PBS group, ^#^*P* < 0.05, ^##^*P* < 0.01, and ^####^*P* < 0.0001 compared to HDM-PBS group. Statistical significance of differences was tested by use of One-Way ANOVA and *post-hoc* Bonferroni's multiple comparisons test.

Oral administration of 20 mg/kg TP, different doses of LGG (10^5^ or 10^7^ cfu/mouse), and their combination as a synbiotic mixture (containing 10^5^ cfu/mouse of LGG and 20 mg/kg TP) could significantly reduce the concentration of IL-5 in the lung homogenates (Figure 4A). There was also a positive correlation between the IL-5 concentration and number of eosinophils in the BALF (Figure 4B, r = 0.9276, p = 0.01). In contrast to Budesonide, the synbiotic mixtures and TP could considerably diminish IL-13 levels in the lung homogenates compared to HDM-PBS group as well (Figure 4C). The chemokine CCL17, which contributes to the Th_2_ cell recruitment in asthma, was elevated in HDM-PBS group compared to PBS-PBS group (p < 0.05). Both TP and synbiotic (10^5^ cfu/mouse of LGG and 20 mg/kg TP) significantly suppressed the chemokine level and the same trend was observed for 10^5^ LGG (p > 0.05) (Figure 4D). Sensitization with HDM increased the concentration of IL-33, a Th_2_-driving mediator, in the lung homogenates. All treatments tended to suppress IL-33 levels in the HDM groups which was not significant (Figure 4E). There was a notable total IgE increase in the HDM-PBS compared to PBS-PBS mice which was suppressed significantly in the group treated with synbiotic (HDM- LGG E5) (p < 0.05) (Figure 4F) (1–4).

### Effect of Oral Administration of LGG, TP, and LGG-TP on Relieving of Maximum Fluorescent Intensity of Th2 and T17 in Splenocytes

**Figure 5:**
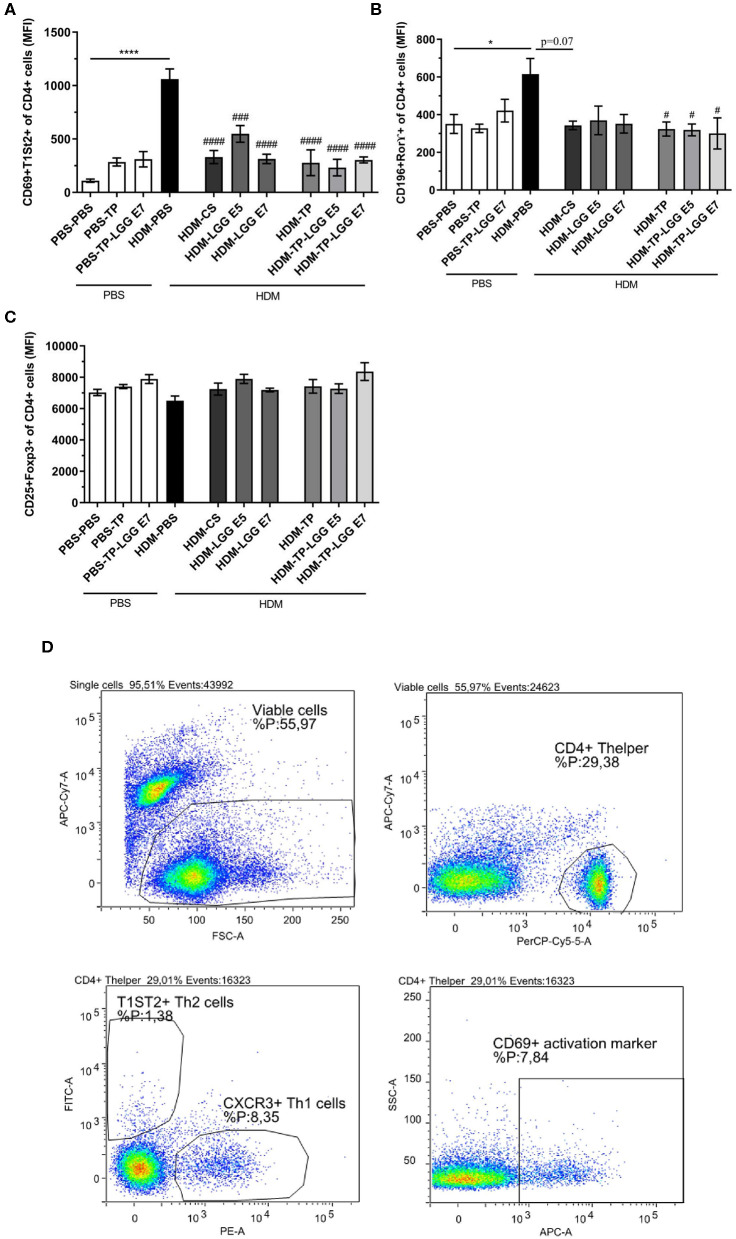
The flowcytometry diagram of T cells subsets. Splenocytes T cell subsets. **(A)** The MFI of Th2 cells (CD69^+^ T1ST2^+^ of CD4^+^ cells), **(B)** Th17 cells (CD196^+^ RORγ+ of CD4^+^ cells), **(C)** regulatory T cells (Tregs) (CD25^+^FoxP3^+^ of CD4^+^ cells) was analyzed in spleen cell suspensions, and **(D)** gating strategy. Values were reported as maximun flourscence intensity (MFI). Data are shown as mean ± *SEM, n* = 6 mice/group. *P < 0.05 and ****P < 0.0001 compared to PBS-PBS group, ^#^*P* < 0.05, ^###^*P* < 0.001, and ^####^*P* < 0.0001 compared to HDM-PBS group. Statistical significance of differences was tested by use of One-Way ANOVA and *post-hoc* Bonferroni's multiple comparisons test.

Splenocytes were analyzed for T cell populations (Figure 5D). The abundance of Th2 and Th17 was significantly increased in asthmatic mice compared to control group (Figures 5A,B). All of the treatments significantly relieved the frequency of Th2 cells. This result is in accordance with the efficiency of LGG, TP and synbiotics in reducing CCL17 chemokine which is implicated in attracting Th2 cells in airways (Figure 4D). It is also elicited from the results that the synbiotics, also TP could significantly decrease the intensity of RORγ^+^CD4^+^Th17 cells. The attenuation of Th2 and Th17 cells by pro- pre- and synbiotics was not followed by a shift toward a more Th1 cells immune response (Supplementary Figure 1). The results also show the CD25^+^Foxp3^+^Treg tended to increase in the synbiotic groups compared to asthmatic group but this was not significant (Figure 5C). The entire of gating strategy has been displayed in Supplementary Figure 2.

### Superior Effect of Synbiotic in Comparison With Probiotic and Prebiotic in Alleviating the Inflammation

According to the results, the synbiotic significantly started to suppress airway hyperresponsiveness from a lower concentration of methacholine (Figure 2).

**Figure 6:**
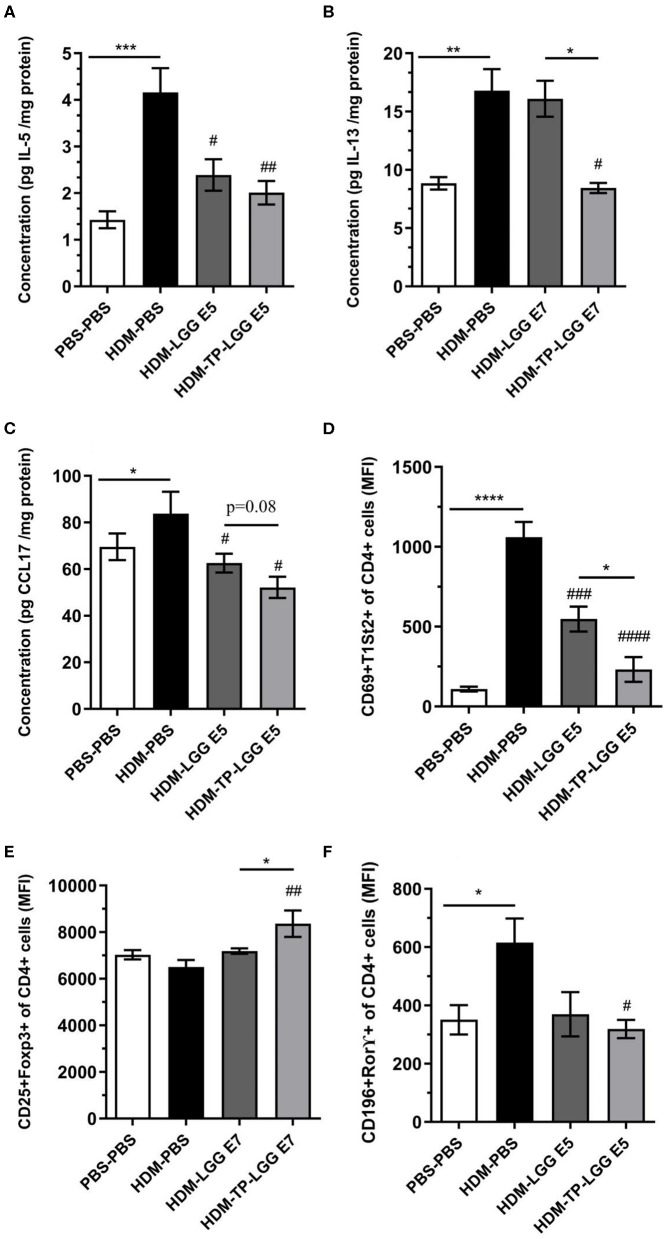
Superior effect of Synbiotic than either of probiotic and prebiotic measurement. IL-5 **(A)**, IL-13 **(B)**, CCL17 **(C)**, CD4+Th2 cells **(D)**, CD25^+^FoxP3^+^Treg **(E)**, and CD196^+^ RORγ+ Th17 **(F)**. Values were reported as maximun flourscence intensity (MFI). Data are shown as mean ± *SEM, n* = 6 mice/group. *P < 0.05, **P < 0.01, ***P < 0.001, and ****P < 0.0001 compared to PBS-PBS group, ^#^*P* < 0.05, ^##^*P* < 0.01, ^###^*P* < 0.001, and ^####^*P* < 0.0001 compared to HDM-PBS group. Statistical significance of differences was tested by use of One-Way ANOVA and *post-hoc* Bonferroni's multiple comparisons test.

Besides, the mice received TP (HDM-TP) or the synbiotic combination with 10^5^ cfu/mouse LGG (HDM-TP-LGG E5) decreased IL-5, IL-13, and CCL17 (Figures 4A,C,D) even more than other treatments. It is also interpreted from the results that the synbiotics (HDM-TP-LGG E5 or HDM-TP-LGG E7) clearly displayed a superior effect than the probiotics to reduce the concentrations of Th2-mediated cytokines such as IL-5 and IL-13 and DC-mediated chemokine CCL17 (Figure 6). This reduction was accompanied by downregulation of activated Th2 cells (CD69^+^T1ST2^+^T cells) and Th17 (CD169^+^Ror⋎^+^ cells). It is also explicit that the synbiotic (HDM-TP-LGG E7) was able to significantly elevate the CD25^+^Foxp3^+^Treg compared to probiotic (HDM-LGG E7).

## Discussion

In the present study, we demonstrated for the first time, that the oral administration of *Lactobacillus rhamnosus* GG, TP, and their combination alleviated the allergic airway inflammation in HDM-induced murine model of asthma. However, all of the treatments (probiotics, prebiotics, and synbiotics) showed suppressive effects on lung function and airway inflammation along with Th2 related cytokines, Th2, and Th17 cells, but we illustrated synbiotic works more effective in comparison with other treatments alone.

Recent studies have consistently begun to elucidate the interaction between gut microbiome function and the immune system response. These researches invested to discover the possible mechanisms by which the resident bacteria can modulate Th2 allergic immune response. However, little is known about the contribution of the molecules secreted by probiotic bacteria (such as SCFA) to act as immune regulators. There is an interplay between prebiotics and probiotics, for example, prebiotics are able to support probiotics growth and lifespan. These findings highlight the need for further investigation on synbiotic mixtures (11–13, 15, 23).

Airway hyperresponsiveness (AHR) is one of the key clinical features of asthma. In this study, HDM-LGG E5 and TP could reduce AHR individually, however, the synbiotic mixture (HDM-TP-LGG E7) demonstrated more potent suppressive effects. In fact, the synbiotic mixture started a reduction of airway hyperresponsiveness in the lower concentration of methacholine (12.5 mg/ml) with a greater impact (*p* < 0.02). This indicates that *Lactobacillus rhamnosus* GG and TP might be able to synergize each other. Moreover, as a standard treatment budesonide treatment showed a decreasing trend in mitigating lung resistance (Figure 2).

The observed contribution of the probiotic, prebiotic and synbiotic is in line with Verheijden et al. who found that the administration of long-chain fructooligosaccharide (lcFOS) combined with *Bifidobaterium breve* M-16V suppressed lung resistance and airway inflammation in allergic mice (22). Vos et al. also showed that a specific oligosaccharide mixture containing short chain galactooligosaccharide (scGOS) and lcFOS (scGOS-lcFOS) could alleviate the lung resistance and BALF inflammatory cells in the ovalbumin (OVA)-induced model of asthma (24). The same combination (scGOS-lcFOS) together with *Bifidobaterium breve* M-16V was used by Sagar et al. (25). They demonstrated that the synbiotic mixture significantly reduces the percentage of BALF total inflammatory cells and eosinophils in a murine model of chronic asthma, which is consistent with our findings. In another study, combination of immunofortis (prebiotic mixture) and *Bifidobaterium breve* M-16V could dampen anaphylactic symptom scores and allergic skin response, which was stronger compared to the individual effects of the pro- and prebiotic alone. The so-called immunofortis and *Bifidobaterium breve* M-16V could also reduce asthma-like manifestations in infants with atopic dermatitis (26). There is a number of studies related to the anti-inflammatory potential of *Lactobacillus rhamnosus* GG and *Bifidobaterium breve* M-16V. Both strains, as well as synbiotic combination of *Bifidobaterium breve* M-16V and scGOS-lcFOS decreased eosinophils and neutrophils in BALF in ovalbumin-exposed mice (1, 25, 26). LGG alone or along with the other bacterial species and prebiotics (galactooligosaccharides, fructooligosassharides) effectively relieved ovalbumin-induced asthma in mice. Given the striking effect of LGG on lung function (2, 27, 28), its combination with turmeric even unveiled more potent effects than any of the individuals. In our study, inhibition of AHR by the synbiotic mixture was associated with the reduced inflammatory cells and cytokines, which might be due to flavonoid components in turmeric possessing powerful anti-allergic properties (19, 29, 30).

In most asthmatic patients, the main cells infiltrated in the lungs are eosinophils, neutrophils, and lymphocytes (18, 31), accumulating in the bronchioles and augment airway inflammation. Among the inflammatory markers involved in asthma, eosinophils orchestrate the paramount inflammatory responses, including airway hyperresponsiveness, mucus secretion of epithelial cells, and allergic cytokines production. Recent data show that eosinophils also participate in airway remodeling (18, 32). We showed that the total inflammatory cells in the BALF were significantly increased in HDM–PBS asthmatic mice compared to PBS–PBS control mice. Oral administration with LGG, TP, and synbiotic mixture markedly decreased the total inflammatory cell numbers into BALF. However, TP with LGG (synbiotic) did not really show a superior effect relative to LGG or TP alone. Probably, a maximum plateau is reached, and a further decrease might be possible in combination with glucocorticosteroids. It is known that there is a correlation between reduction of inflammatory cells in the lung and suppression of Th2 associated cytokines (33–35). Consistent with this, we found the reduction of eosinophils in mice treated with TP, LGG, and synbiotic mixture, which was associated with a decrease in IL-5, IL-13, and IL-33 production.

Allergic asthma is related to a Th1/Th2 imbalance with increased Th2 cytokines production (18). HDM triggers inflammation in airway epithelial cells (AECs), which consequently activates pattern recognition receptors (PRRs), in particular, Toll-like receptors (TLRs) (36, 37). Interaction between HDM and AECs is the central point of sensitization phase recruiting inflammatory cells to the airway submucosa. Subsequent exposure to HDM (challenge phase) augments the production of IL-33, CCL17, TSLP, IL-25, and different chemokines causing clinical manifestations.

Many investigations showed that the concentration of CCL17, IL-33, CCL20, and CCL22 were increased in the airways of asthmatic patients compared to healthy individuals (32, 38–40). The concentration of IL-5 and CCL17 showed a decreasing trend in synbiotic (HDM-TP-LGG E5) and prebiotic group. CCL17 is mainly responsible for recruiting and activating neutrophils to the lungs (41). The falling trend of neutrophils in the BALF and CCL17 in the lung homogenates was found in asthmatic mice treated with synbiotic (HDM-TP-LGG E5) and prebiotic alone compared to the HDM-PBS group (Figures 3E, 4D). The Synbiotic with 10^5^ cfu/ml of LGG displayed a better suppressive effect than the probiotic (HDM-LGG E5) while the other synbiotic could not demonstrate the same effect (data not shown) (Figure 6C). This phenomenon can be attributed to the so-called compatibility of probiotic and prebiotic which seems to be a key factor in synbiotic proficiency. IL-13 was another pivotal mediator which the synbiotics could impact on. In this regard, HDM-TP-LGG E7 was significantly more effective than the probiotic in lowering IL-13 (Figure 6B).

IL-33 activates DCs and attracts Th2 cells in the course of Th2 responses (35, 42, 43). Clinically, in the biopsies of asthmatic patients, the vigorous increase of IL-33 has been observed compared to healthy people (44). Here we also showed a high level of IL-33 in the lung homogenates of asthmatic mice compared to non-asthmatic groups (*p* < 0.05). The synbiotics showed a reduction trend of IL-33 level rather than other treatments, however, the reduction was not statistically significant (Figure 4E).

The exact underlying mechanisms by which TP, LGG or synbiotic ameliorate inflammation remain unclear and need further exploration. In this study, we made an attempt to review some of the known characterized mechanisms. Several pieces of evidence indicate that the dysbiosis in the intestinal microbiota composition is associated with respiratory disorders. In many studies the considerable reduction in the abundance of bifidobacteria and lactobacilli genera has been found in asthmatic patients compared to healthy controls (45). The gut dysbiosis also encourages inflammation through the growth of Enterobacteriaceae and the decrease of Lactobacilli and Lactococci (46). Colestridia, *Haemophilus, Streptococcus*, and *Moraxella* species have also been correlated with elevated risk of asthma exacerbations (47). Some bacterial species especially *Lactobacillus rhamnosus* GG can stimulate naïve T cells to be differentiated to peripheral Treg (48). The commensal bacteria along with lactobacilli and bifidobacteria are able to turn down Th2 cell differentiation by producing special metabolites such as short-chain fatty acids (26, 45). In line with these studies, our findings show that the probiotic could shift the naïve T cells to Treg, however the synbiotic indicated more potency to increase Tregs intensity (Figure 6E). Besides, the ability that the synbiotic was more remarkable in downregulating Th2 cells compared to either asthmatic group or probiotic (Figure 6D). Several evidences show the effect of gut microbiota and herbal components are due to the SCFA production (7, 49–51). Many studies also indicate that the enhanced effectiveness of synbiotic above either of the constituents (probiotic and prebiotic) is due to the production of SCFA, this implies that there is a need to measure the concentration of SCFA produced by pro-, pre- and, synbiotic in further studies to unravel the mechanism (52).

One of the mechanisms by which SCFAs regulate the immune responses is to enhance the CD103^+^DCs of MLNs and subsequently increase the activity of retinal dehydrogenase 2(RALDH2) in CD103^+^ DCs. The conversion of Vitamin A to retinoic acid which is mediated by RALDH2 stimulates Treg cells generation (48). In line with this study, our combination therapy (HDM-TP-LGG E7) significantly increase Treg compared to the TP and LGG alone which is presumably due to the concentration of SCFA produced by LGG when is together with turmeric. The other mechanism could involve in G protein–coupled receptor 41 (GPR41, also called free fatty acid receptor 3 or FFAR3) (53). SCFA may elicit their regulatory functions through binding to this protein which may impair the capacity of DCs to stimulate Th2-mediated immune responses (54). Given that the production of SCFA propionate, acetate and butyrate depends on indigestible carbohydrate fermentation, the role of prebiotics in immune response regulation are revealed.

Th17 cells, characterized by the retinoic acid-related orphan receptor ⋎ (ROR ⋎) marker, which is a main transcription factor mediating the Th17 differentiation, can stimulate tissue inflammation, and neutrophil recruitment (55). Recent findings have suggested that Th17 cells and its mediated cytokines were implicated in the pathogenesis of allergic asthma. The results indicate our synbiotics and prebiotic significantly reduced CD196^+^ROR ⋎^+^Th17 cells frequency compared to asthmatic group (Figures 5B, 6F). The marker CD196 also called chemokine receptor 6 (CCR6), and its ligand CCL20, contributes to the recruitment of Th17 cells and Th2 cells to the injured tissue and specially deal with the asthma exacerbation. Since the airway responsiveness was relieved in CCR6-deficient mice, CCL20-CCR6 axis could be a putative approach for the asthma therapy (56).

**Figure 7:**
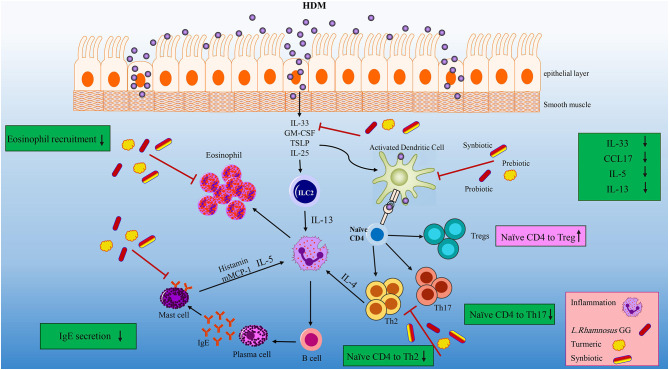
Overview of the effects of the LGG, TP and synbiotic treatment on HDM-induce murine model of asthma. After the initial exposure to HDM IL-33, GM-CSF, TSLP and IL-25 is released by damaged tissue, and IL-33 activate DCs and ILC2. CCL17 are proliferated by activated DC, which can polarize naïve T cells into Th2 cells. LGG, TP or combination therapy tended to reduce CCL17 and IL-33 concentrations, leading to decrease DCs activation. Beyond their effect on suppressing DC-related cytokine and chemokine, they reduced the differentiation of naïve T cells to Th2 cells significantly. ILC2 cells as well as Th2 cells have the ability to produce IL-4, IL-5, and IL-13. Concentrations of IL-5 and IL-13 were reduced after specifically synbiotics treatment. The number of eosinophils reduced by all treatments specially 10^5^ cfu/ml LGG. All treatments could significantly reduce the Th2 and Th17 frequency. This reduction was more remarkable in HDM-TP-LGG E5 compared to asthmatic and probiotic group. On the other hand, the Treg frequency showed an elevation in HDM-TP-LGG E7 compared to asthmatic and probiotic group.

The first line of allergic inflammation is the elevation of IL-33, which is mainly expressed by HDM injured lung epithelial cells (Figure 7). IL-33 is also a chemo-attractant for Th2 cells to the inflammation site (35, 42, 43). Synbiotic intervention tended to reduce the concentration of IL-33. The IL-33 secretion along with the allergen stimulation can activate ILC2 and DCs. Activated DCs are able to polarize naïve T helpers to Th2 in the CCL17 saturated environments (57). Our treatments specially HDM-TP-LGG E5 proficiently mitigate the concentration of CCL17 leading to the decreasing of Th2 frequency (Figure 7). On the other hand, ILC2 together with Th2, secrete IL-13, IL-5, and IL-4 which in turn differentiates B cells to IgE producing plasma cells (58, 59). Probiotic, prebiotic, and synbiotic interventions significantly lessened IL-5 and IL-13 production. This effect was more striking in synbiotic groups (Figure 7). Beyond this effect on Th2-mediated cytokines, the combination therapies also mitigate the intensity of CD196^+^RORγ^+^Th17 cells (Figure 7) which is known to be elevated in asthmatic peoples (57). Our treatments specially probiotics reduces the number of eosinophils in BALF. As far as we know, the mechanism of our pro-, pre, and synbiotic in Th2 suppression is independent to the Th1 since HDM sensitization and challenge did not affect the frequency of Th1 and even combination treatment could not affect it.

Our study not only strengthens the previous evidence regarding the beneficial effects of probiotics and prebiotics in the suppression of allergic responses but also suggests that their combination (a synbiotic) might have a superior effect than any of each treatment alone. In this study, we sensitized the mice with HDM and then treated with PBS, LGG, TP, and synbiotics orally during the exposure of HDM and evaluated the lung hyperresponsiveness to the different doses of methacholine. We also measured the number of inflammatory cells in the BALF and concentration of cytokines and a chemokine which are responsible for allergic manifestation.

The present study has assessed the modulatory effects of pre, pro, and synbiotic on allergic airway inflammation at the cellular level. However, more broadly, research is needed to determine the signaling pathways by which the combination therapy suppresses the inflammatory cascade. The exploring short chain fatty acids produced by LGG with/without TP, characterization the probable components released by LGG like SCFA, and interaction between TP and LGG also need to be considered as further investigations. It is also worthwhile to optimize the repetition times and the duration of the treatment that lasts long effect on the body. In addition, the other factors which determine the optimal efficacy of a synbiotic, are 1) the duration of prebiotic and probiotic reciprocal interaction and 2) the ability of probiotic to be implanted into the gut, thus optimizing these factors can be highly essential for development of food-based remedy.

## Data Availability Statement

The raw data supporting the conclusions of this article will be made available by the authors, without undue reservation, to any qualified researcher.

## Ethics Statement

This study was carried out in strict accordance with the recommendations in the Guide for the Care and Use of the Dutch Committee of Animal Experiments (Utrecht, the Netherlands). The protocol was approved by the Committee on the Ethics of Animal Experiments of the Utrecht University (Protocol Number: 1080020174426). All surgery was performed under sodium pentobarbital anesthesia, and all efforts were made to minimize suffering.

## Author Contributions

FG, AZ, and GF conceived and designed the project. FG, TL-M, and IA performed the experiments. FG, SB, TL-M, and IA analyzed the data. FG and AZ wrote the manuscript. SS-Z, SB, GF, AZ, and FG reviewed and edited the manuscript. FG, TL-M, and IA performed/assisted with experiments. FG performed the microbiota analysis. GF contributed reagents/materials/analysis tools. GF and SS-Z obtained the funding and ST helped out with technical assistance and helpful discussions.

## Conflict of Interest

The authors declare that the research was conducted in the absence of any commercial or financial relationships that could be construed as a potential conflict of interest.
