# Supplementary material for: Crude Turmeric Extract Improves the Suppressive Effects of Lactobacillus rhamnosus GG on Allergic Inflammation in a Murine Model of House Dust Mite-Induced Asthma
Source: Front Immunol. 2020 Jun 4;11:1092. doi: 10.3389/fimmu.2020.01092 (PMC7287160; doi:10.3389/fimmu.2020.01092)
Supplement: Supplementary file 1 [file Data_Sheet_1.PDF]

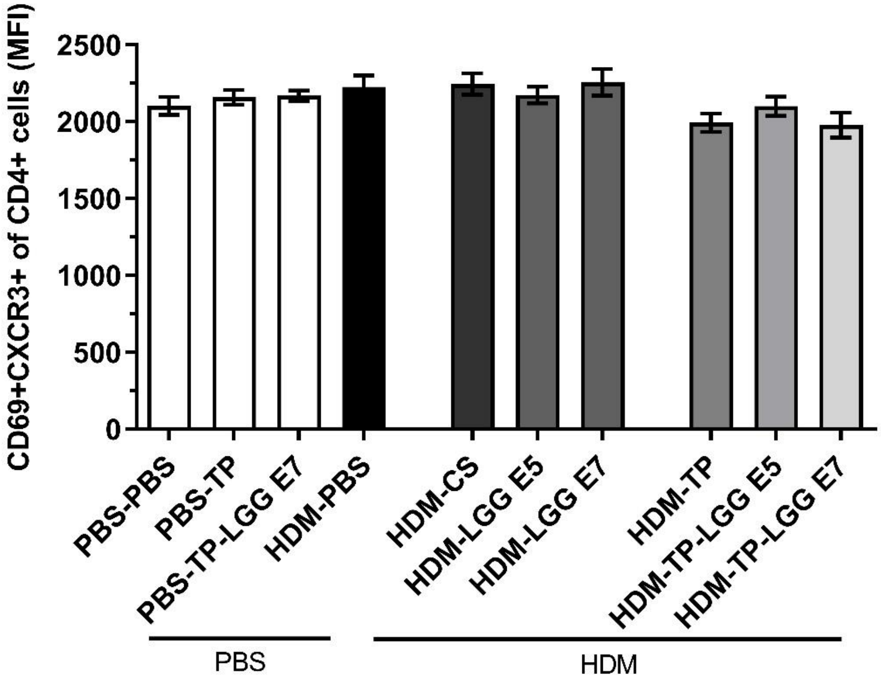

Supplementary Figure 1: The flowcytometry diagram of Th1 cells. (A) The MFI of Th1 cells (CD69$^+$ CXCR3$^+$ of CD4$^+$ cells was analyzed in spleen cell suspensions. Values were reported as maximun flourscence intensity (MFI). Data are shown as mean ± SEM, n = 6 mice/group.
