# Supplementary figures and images for: Crude Turmeric Extract Improves the Suppressive Effects of Lactobacillus rhamnosus GG on Allergic Inflammation in a Murine Model of House Dust Mite-Induced Asthma
Source: Front Immunol. 2020 Jun 4;11:1092. doi: 10.3389/fimmu.2020.01092 (PMC7287160; doi:10.3389/fimmu.2020.01092)

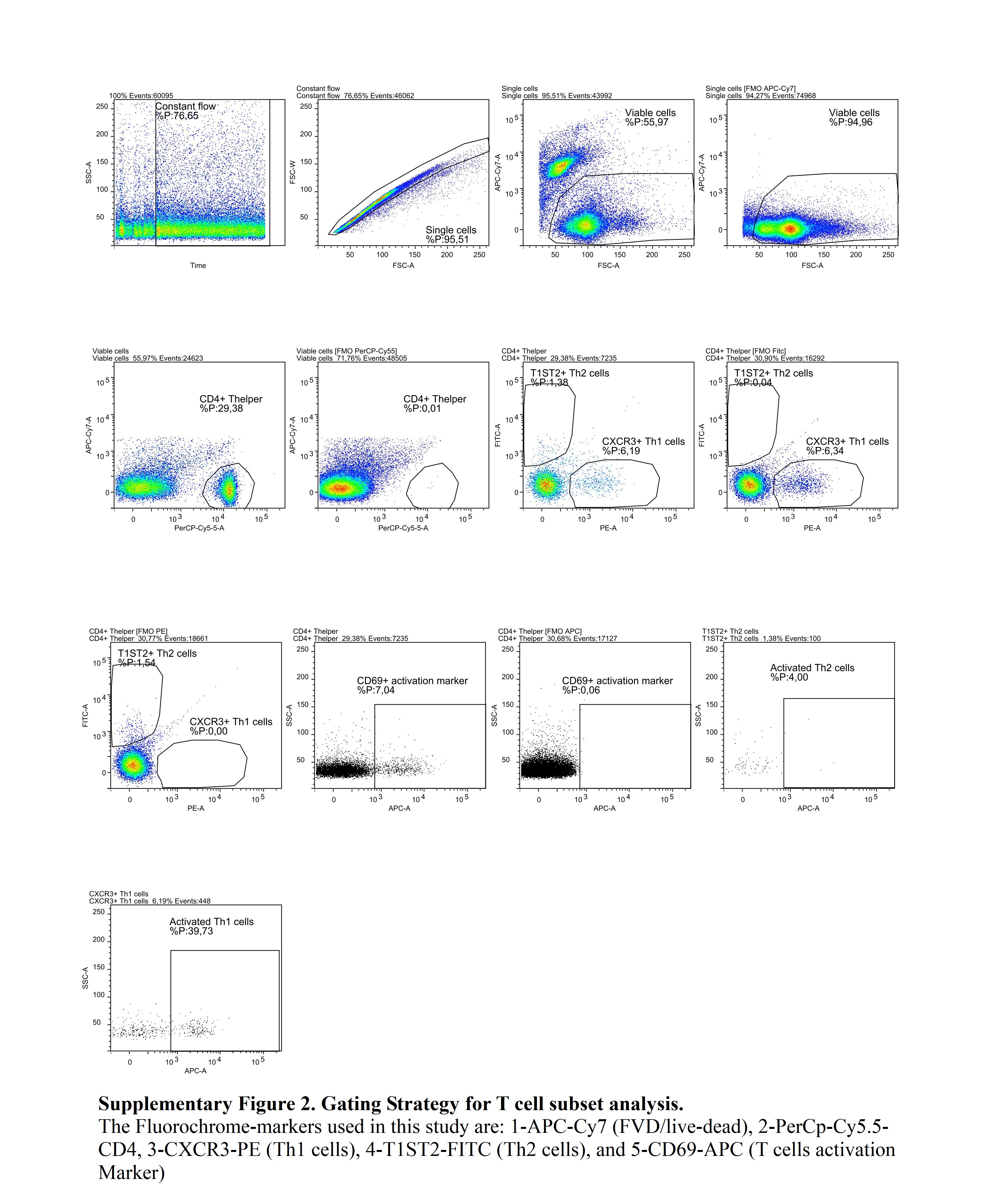

Supplement: Supplementary file 2 [file Image_1.JPEG]
